# Green Flexible Polyurethane Foam as a Potent Support for Fe-Si Adsorbent

**DOI:** 10.3390/polym11122011

**Published:** 2019-12-04

**Authors:** Afiqah Ahmad, Siti Nurul Ain Md. Jamil, Thomas Shean Yaw Choong, Abdul Halim Abdullah, Mohd Sufri Mastuli, Nurhanisah Othman, NurNazurah Jiman

**Affiliations:** 1 Department of Chemistry, Faculty of Science, Universiti Putra Malaysia, UPM Serdang 43400, Selangor, Malaysia; afiqahahmad95@yahoo.com (A.A.); halim@upm.edu.my (A.H.A.); hanisahlab@gmail.com (N.O.); nnazurahjiman@gmail.com (N.J.); 2 Centre of Foundation Studies for Agricultural Science, Universiti Putra Malaysia, UPM Serdang 43400, Selangor, Malaysia; 3 Department of Chemical and Environmental Engineering, Faculty of Engineering, Universiti Putra Malaysia, UPM Serdang 43400, Selangor, Malaysia; csthomas@upm.edu.my; 4 Institute of Advanced Technology, Universiti Putra Malaysia, UPM Serdang 43400, Selangor, Malaysia; 5 Centre of Nanomaterials Research, Institute of Science, Universiti Teknologi MARA, Shah Alam 40450, Selangor, Malaysia; mohdsufri@uitm.edu.my

**Keywords:** flexible foam, polyurethane, support, wastewater treatment, dye adsorption, palm oil, iron silica, methylene blue, reusability

## Abstract

This paper describes the preparation, characterisation, and potential application of flexible palm oil-based polyurethane foam (PUF) as a support for iron-silica (Fe-Si) adsorbent. Fe-Si/polyurethane composite (Fe-Si/PUC) was prepared by impregnating Fe-Si adsorbent onto the surface of PUF by using a novel immersion-drying method. Morphological analysis of Fe-Si/PUC proved that Fe-Si was successfully impregnated onto the surface of PUF. Compression test and thermogravimetric analysis were carried out to determine the flexibility and thermal stability of Fe-Si/PUC, respectively. The Fe-Si/PUC removed 90.0% of 10 ppm methylene blue (MB) from aqueous solution in 60 min. The reusability study showed that Fe-Si/PUC removed 55.9% of MB on the seventh cycle. Hence, the synthesis of Fe-Si/PUC opens up a new path of implementing palm oil-based PUF to assist in the recovery of an adsorbent for environmental clean-up. The mechanism of physical interaction during the impregnation of Fe-Si adsorbent onto PUF was proposed in this paper.

## 1. Introduction

Water pollution is a serious problem in the whole world and it is caused by the spillage of chemicals and dyes. The dyes which originated from textile industry has contributed 22.0% of the total volume of industrial wastewater in Malaysia [1]. According to a study that was conducted on batik industry in Kelantan, Malaysia, small batik premises do not have proper waste disposal management and the wastewater was directly discharged into the river [2]. Untreated and uncontrolled disposal of dyes into the water system has become a significant concern, as it leads to water contamination. Water pollution poses threats towards humans and aquatic lives; hence, treatment and prevention need to be taken.

Methylene blue (MB) is a cationic dye that has been widely used in textile industries and medical applications. However, an excessive dosage of MB was reported to have adverse effects; e.g., cardiac arrhythmias and coronary vasoconstriction in human (for dosage more than 2.00 mg/kg) [3]; and, mortality effect in fish and crustaceans (PAN Pesticide Database). Practices for treating dye effluent in wastewater ranges from physical, chemical, and biological methods. Some of the chemical methods include adsorption, advanced oxidation, and photocatalytic degradation [4,5,6].

Adsorption technique is one of the commonly used techniques for water treatment due to the cost effectiveness and high adsorption rate [7]. For dyes removal, several adsorbents have been widely used, such as graphene oxide [8], activated carbon [7], and modified silica [9]. Iron-silica (Fe-Si) has been reported to act as both adsorbent and catalyst. Fe-Si has a high surface area, low toxicity, and is chemically stable over a wide range of pH [10,11]. Fe-Si has been previously applied as an adsorbent for the removal of heavy metal ions, dye, and volatile organic compounds [9,10,11,12,13]. Chromium (III) adsorption study was conducted by Egodawatte et al. while using iron oxide/mesoporous silica nanocomposites functionalised with the aminopropyl group. Egodawatte et al. reported that the nanocomposite showed high adsorption affinity for positively charged compounds and low affinity for negatively charged compounds. The adsorption capacity for chromium was 2.08 mmol/g [10]. Zeng synthesised silica-containing iron (III) adsorbent and reported that the adsorbent with higher iron content has higher capacity in removing arsenic [13]. Popova et al. synthesised iron-functionalised silica nanoparticles as adsorbent and catalyst for toluene oxidation. Popova and team also reported that high iron content leads to high toluene oxidation, while the adsorption study found that the adsorption capacity of toluene was 34.90 mg/100.00 g of the sample [12]. Recently, Liu and the team reported the application of iron incorporated in mesoporous silica film in adsorbing and degrading MB. The thin film managed to adsorb (96.9%) and degrade (98.6%) MB in the presence of hydrogen peroxide, in 5 h. However, application of adsorbent alone might lead to secondary pollutants, where the adsorbent could leach into the water and hardly be retrieved. Therefore, in this study, Fe-Si adsorbent is embedded on a support to enhance its efficacy in adsorbing MB.

Polyurethane foam (PUF) is cost-effective, as it is synthesised from a palm oil-based polyol. Increased price and limited supply of petroleum-based products caused consumers to turn to renewable and sustainable sources, such as palm oil. Palm oil-based polyurethane comes in different forms; e.g., rigid and flexible foams, film, and composite [14,15,16,17]. PUF is commonly used in construction and automotive industries [18,19,20]. In recent years, the application of PUF was broadened to wastewater treatment and it has been frequently used as a support for adsorbents and catalysts [21,22,23,24]. Commercial and in-house PUF provide high surface area for sorbent or catalyst impregnation, and it facilitates the recovery process of adsorbent and catalyst in water treatments.

Different methods for adsorbent or catalyst impregnation onto PUF were reported, e.g., via in-situ immobilisation, coating, and surface pre-treatment [21,24,25]. Previously, Sayed & Burham prepared polyurethane foam/organobentonite/iron oxide nanocomposite via in-situ polymerisation of toluene diisocyanate and polyol in the presence of organobentonite/iron oxide for the adsorption of cadmium from an aqueous solution. It was reported that the nanocomposite managed to remove up to 98.5% of cadmium within 60 min. [24]. In another study by Lefebvre et al., silver nanoparticles were impregnated onto polydopamine-coated polyurethane and the composite was utilised for the reduction of MB. They reported that, in the presence of sodium borohydrate, over 95.0% of MB was removed within 25 min. or up to 6 cycles [21]. Recently, Cui et al. reported a method for immobilisation of lipase onto PUF by surface pre-treatment with hydrochloric acid. They found that lipase immobilised on hydrochloric acid-modified PUF has higher enzyme activity (8000 U/g), as compared to lipase immobilised on untreated PUF (5300 U/g). The immobilised lipase was also reported to have high reusability (up to 18 times) with a conversion rate of more than 90.0% in the production of Vitamin A palmitate [25]. Based on the previous methods reported, surface pre-treatment is the most suitable method for the embedment of Fe-Si onto the surface of PUF. By having Fe-Si adsorbent embedded on PUF, it is hoped to ease the MB removal process. In addition, Fe-Si/polyurethane composite (Fe-Si/PUC) contributes to the full recovery/minimal loss of Fe-Si adsorbent, as compared to Fe-Si working alone, which easily leached into the water.

Therefore, in this paper, a novel method for the impregnation of Fe-Si adsorbent onto the surface of in-house flexible palm oil-based PUF is described. The flexible PUF was utilised as a support to facilitate the adsorbent recovery process without reducing the performance of the adsorbent itself. Another advantage for having PUF as a support is to avoid a secondary pollution, such as the formation of toxic sludge and sedimentation contributed by the uncovered adsorbents. A novel approach was applied to synthesise the Fe-Si/PUC, which includes less organic solvent use. Fe-Si adsorbent with high surface area and thermal stability is synthesised by the sol-gel method and characterised. The stability of Fe-Si/PUC, the proposed impregnation interaction, and the performance of Fe-Si/PUC in MB removal are further discussed. To date, the preparation of Fe-Si adsorbent that is embedded on the surface of flexible PUF has not been reported elsewhere. The fact that the synthesis method is simple yet novel, the adsorbent is cost effective and environmentally friendly fit the purpose of this paper, which is to produce a green adsorbent that is supported by a natural-based support for dye removal.

## 2. Materials and Methods 

### 2.1. Materials

Palm oil polyol was obtained from Malaysian Palm Oil Board (MPOB) (Bangi, Malaysia) with hydroxyl value of 220.00 mg of KOH/g of polyol. Tolylene-2,4-diisocyanate (TDI) 85.0%, polypropylene glycol (PPG) 1200, tetraethyl orthosilicate (TEOS) 98.0%, iron (III) perchlorate hydrate Fe(ClO_4_)_3_·H_2_O, and methylene blue (MB) were purchased from Sigma Aldrich (Gillingham, UK). The catalyst used was N,N-dimethylcyclohexylamine (DMCHA), purchased from Merck (Darmstadt, Germany) and the surfactant polysiloxane 1280 was purchased from Dow Corning (Auburn Hills, MI, USA). Ethanol 95.0%, sodium hydroxide (NaOH), hydrochloric acid (HCl), and ammonia (NH_4_OH) solution were purchased from R&M Chemicals (Semenyih, Malaysia).

### 2.2. Synthesis of PUF

Briefly, 15.00 g of palm oil polyol, 8.00 g of PPG, 0.60 g of distilled water, 0.30 g of DMCHA, and 45 µL of surfactant were mixed in a paper cup by using overhead stirrer at 500 rpm until the mixture turned cloudy. 12.00 g of TDI was then poured into the mixture and stirred at 500 rpm for 15 s. PUF produced was left to rise and cured at room temperature for 24 h. The synthesised PUF has a porosity (*Ɛ*) of 0.32.

### 2.3. Synthesis of Fe-Si adsorbent

Fe-Si was synthesised by using the modified sol-gel method followed by calcination [11]. Fe(ClO_4_)_3_·H_2_O (1.0 M) was dissolved in 100.00 mL of ethanol (1.0 M), followed by addition of TEOS (1.0 M). The reaction mixture was stirred at 300 rpm, 80 °C for 2 h. Sol-gel formation was induced by the addition of 50.00 mL of NH_4_OH (3.0 M) and 50.00 mL of distilled water, dropwise. The reaction was continued for another 2 h at 500 rpm, 80 °C. The sol-gel was transferred into a beaker and then dried at 110 °C for 24 h. The resulting product was washed with distilled water (at least three times) and dried at 110 °C for 24 h. The brown powder obtained was calcined in a muffle oven at 550 °C for 4 h.

### 2.4. Impregnation of Fe-Si onto PUF (Fe-Si/PUC) 

0.50 g of Fe-Si was added into a beaker containing 10.00 mL of ethanol (95.0%). The mixture was sonicated for 10 min. 0.20 g of PU foam (2.0 cm × 2.0 cm × 1.0 cm) was immersed and gently stirred in the suspension until the adsorbent fully covered the foam surface. The resulting composite was dried at 60 °C for 45 min. Immersion and drying processes were repeated four times to ensure the maximum impregnation of adsorbent. The final product was washed with distilled water to remove excess Fe-Si and dried at 60 °C for 24 h. The amount of Fe-Si impregnated was calculated by using Equation (1) to obtain a percentage deviation of less than 10.0%. Ethanol-treated PUF (PUT) was prepared by immersing PUF in 10.00 mL of ethanol, washed, and dried at 60 °C for 45 min. PUT acts as a control where the surface was treated with ethanol, without the embedment of the Fe-Si.

Percentage of Fe-Si impregnated = ((*m*_f_ − *m*_i_)/*m*_i_) × 100 (1)

where *m*_i_ = initial mass of PUF; *m*_f_ = final mass of impregnated product (Fe-Si/PUC).

### 2.5. Characterisations

Fourier Transform Infrared (FT-IR) spectra were recorded by using BX Perkin Elmer (Akron, OH, USA) with Universal Attenuated Total Reflectance (UATR) technique to identify functional groups that are present in the Fe-Si, PUF, PUT, and Fe-Si/PUC. Morphological analysis and qualitative chemical composition of the Fe-Si, PUF, and Fe-Si/PUC were examined by using field emission scanning electron microscope-energy dispersive x-ray (FESEM-EDX) JEOL JSM-7600F (Tokyo, Japan). Thermogravimetric analysis (TGA/DTG) on Fe-Si, PUF, PUT, and Fe-Si/PUC was carried out to test the thermal stability by using thermogravimetric analyser (TGA/SDTA 851, Mettler Toledo, Columbus, OH, USA), from room temperature to 900 °C with a heating rate of 10 °C min.^−1^. The surface area of Fe-Si was determined by using the Brunauer-Emmett-Teller (BET) method (Micromeritics Instrument Corporation, Model-3Flex, Norcross, GA, USA). X-ray diffraction (XRD) patterns of Fe-Si was measured by using PANalytical X’Pert PRO (Malvern, UK). Water absorption analysis was carried out by immersing 0.20 g of PUF, PUT. and Fe-Si/PUC (2.0 cm × 2.0 cm × 1.0 cm) in a beaker containing 50.00 mL of tap water, respectively, for 24 h. The volume of water uptake was calculated by using Equation (2) Compressive test analysis was done by using Universal Tensile Instron Series 2716 (Norwood, MA, USA) according to standard method BS 4370: Part 1:1988: Method 3. PUF, PUT, and Fe-Si/PUC with dimensions of 4.0 cm × 4.0 cm × 4.0 cm were prepared. The initial foam level was marked and a compression rate of 10 mm/min. was applied. Foams were compressed up to 10.0% of the initial thickness. Foam compressive stress and modulus were recorded and analysed.

Percentage of water uptake = ((*m*_f_ − *m*_i_)/*m*_i_) × 100 (2)

where *m*_i_ = mass of samples before the immersion; *m*_f_ = mass of samples after the immersion.

### 2.6. Preliminary Study of Fe-Si/PUC for MB Removal in Aqueous Solution

A flask containing 100.00 mL of aqueous MB solution (10 ppm) was placed in water bath shaker for continuous stirring. The experiment was carried out at room temperature and then opened to the atmosphere. The initial pH was adjusted to 7 ± 0.05 by using NaOH (0.1 M) or HCl (0.1 M). Fe-Si/PUC (0.10 g Fe-Si loaded) was placed inside the reaction system and then stirred at 100 rpm. Controls were carried out by using PUF (0.20 g), PUT (0.20 g), and Fe-Si (0.10 g) as sorbent. Subsquently, 2.00 mL of samples were taken out at specific time intervals. The concentration of MB was determined by using Shimadzu UV-1280 spectrophotometer (Kyoto, Japan) at 664 nm^−1^. The pH of the solution was not controlled throughout the experiment. Each experiment was done in triplicate with a maximum error of 10.0%.

### 2.7. Reusability Study of Fe-Si and Fe-Si/PUC

Used Fe-Si and Fe-Si/PUC were collected and washed by using distilled water. The samples were dried at room temperature for 24 h. Flasks containing 100.00 mL of aqueous MB solution (10 ppm) were placed in water bath shaker for continuous stirring. The reaction was carried out at room temperature and opened to the atmosphere. The initial pH was adjusted to 7 ± 0.05 by using NaOH (0.1 M) or HCl (0.1 M). Fe-Si (0.10 g) and Fe-Si/PUC (0.10 g Fe-Si loaded) were placed in each flask, respectively. Afterwards, 2.00 mL of samples were taken out at specific time intervals. The concentration of MB was determined by using Shimadzu UV-1280 spectrophotometer (Kyoto, Japan) at 664 nm^−1^. The pH of the solution was not controlled throughout the experiment.

### 2.8. Quantification of Fe-Si Powder and Fe Residual from Fe-Si/PUC during the Reusability Study

The MB solution of each cycle was collected and analysed to determine the amount of total suspended solids (TSS) of Fe-Si powder. TSS analysis was conducted based on the Standard Methods (2017), 2540D. Briefly, the volume of the adsorbed MB solution was measured and then filtered through a pre-weighed filter. The filter was heated to a constant mass at 104 °C and weighed. The concentration of suspended Fe-Si was calculated by dividing the mass of the solid collected by the volume of MB solution. On the other hand, dissolved Fe in the filtered MB solution was determined by using atomic absorption spectrometer (AAS) (iCE 3000 AAS, Thermo Fisher Scientific, Waltham, MA, USA). This study was limited to only quantifying Fe-Si powder and Fe residual. Quantification of Si residual was not carried out, as Si was not listed in the Malaysian Environmental Quality (Industrial Effluent) Regulations 2009.

## 3. Results and Discussion

### 3.1. Impregnation of Fe-Si onto PUF

**Figure 1:**
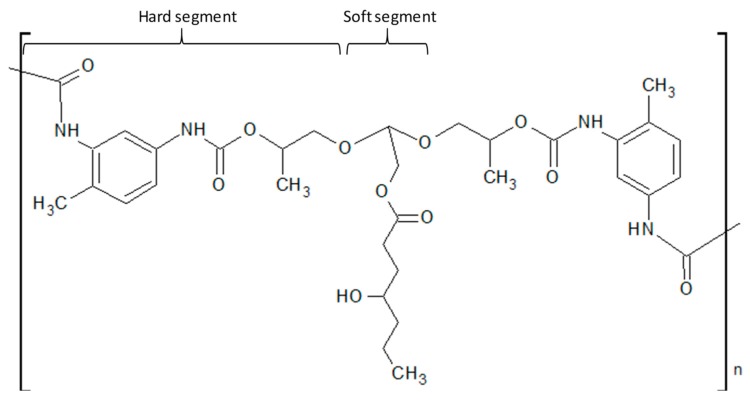
Chemical structure of polyurethane foam (PUF). The hard segment is made up of toluene isocyanate and extended by propylene glycol. The soft segment consists of hydrocarbon chain from palm oil polyol.

The immobilisation of Fe-Si onto the surface of PUF was achieved by physical interaction between Fe-Si and PUF. In the present work, the impregnation was done by immersing the PUF in ethanol containing dispersed Fe-Si. It was observed that the PUF expanded during the immersion. The synthesised PUF (Figure 1) consisted of hard segment (toluene diisocyanate extended with propylene glycol) and soft segment (palm oil polyol). The hard segment contained higher polarity group (–NH–C(O)–) when compared to the soft segment (–COC–). 

The treatment of PUF with ethanol might be responsible in removing oligomers with low molecular weight in the soft segments [26]. The removal of some of the soft segments resulted in the exposure of the hard segment towards Fe-Si. As the hard segment contained polar group, it had a high tendency to form interactions with Fe-Si, which is more electropositive due to the presence of Fe. Note that the PUT and Fe-Si/PUC in this work had good surface polarity due to its excellent performance during water uptake analysis, as discussed in Section 3.6.

Previously, Chen and Ruckenstein reported that the pre-treatment of PU foam with organic solvents resulted in molecular rearrangement of the PU foam surface. The molecular rearrangement was proven by the surface contact angle and Electron Spectroscopy for Chemical Analysis (ECSA) [27]. This indirectly supports the proposed interaction, where the surface of the PUF can be altered with organic solvent for further enhancement.

### 3.2. FT-IR

**Figure 2:**
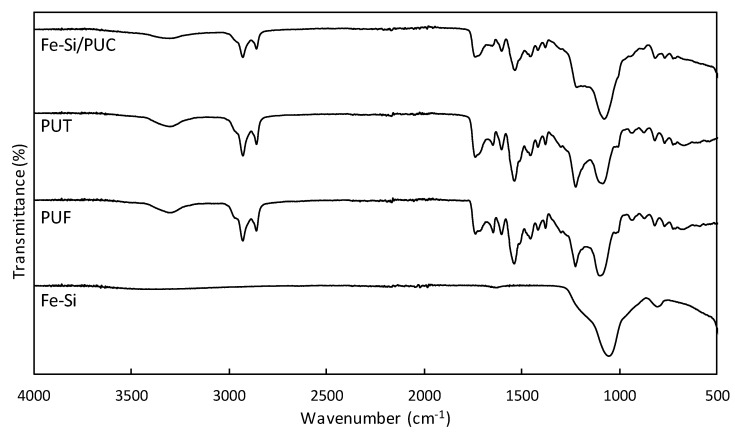
Fourier Transform Infrared (FT-IR) spectra of Iron-silica/polyurethane composite (Fe-Si/PUC), ethanol-treated PUF (PUT), PUF, and Fe-Si.

FT-IR was used to determine the functional group present in Fe-Si, PUF, PUT, and Fe-Si/PUC (Figure 2). Two adsorption bands (1051 cm^−1^ and 802 cm^−1^ wavenumbers) appeared on the Fe-Si spectrum, which correspond to O–Si–O and Si–O–Si stretching vibrations. A similar observation was reported for IR spectrum of silica precursor TEOS [28]. The adsorption bands observed in PUF, PUT, and Fe-Si/PUC spectra were similar. The band at 3294 cm^−1^ corresponded to N–H stretching, while 1533 cm^−1^ was contributed by N–H bending in isocyanate group [16], [29]. The peaks at 2924 cm^−1^ and 2854 cm^−1^ represent aliphatic C–H stretching of the methylene group in TDI. Urethane C=O vibration can be observed at 1734 cm^−1^, C=O stretching at 1220 cm^−1^ in urethane, and C-O vibration at 1097 cm^−1^ [16], [29]. The appearance of absorption bands at 1643 cm^−1^ and 1537 cm^−1^ represent CN–H stretching and bending, respectively [17]. The intensity of peaks that corresponds to C=O stretching (1220 cm^−1^) decreased, while the absorption band that corresponds to C–O vibration (1097 cm^−1^) increased for Fe-Si/PUC when compared to PUF and PUT. 

### 3.3. FESEM-EDX

**Figure 3:**
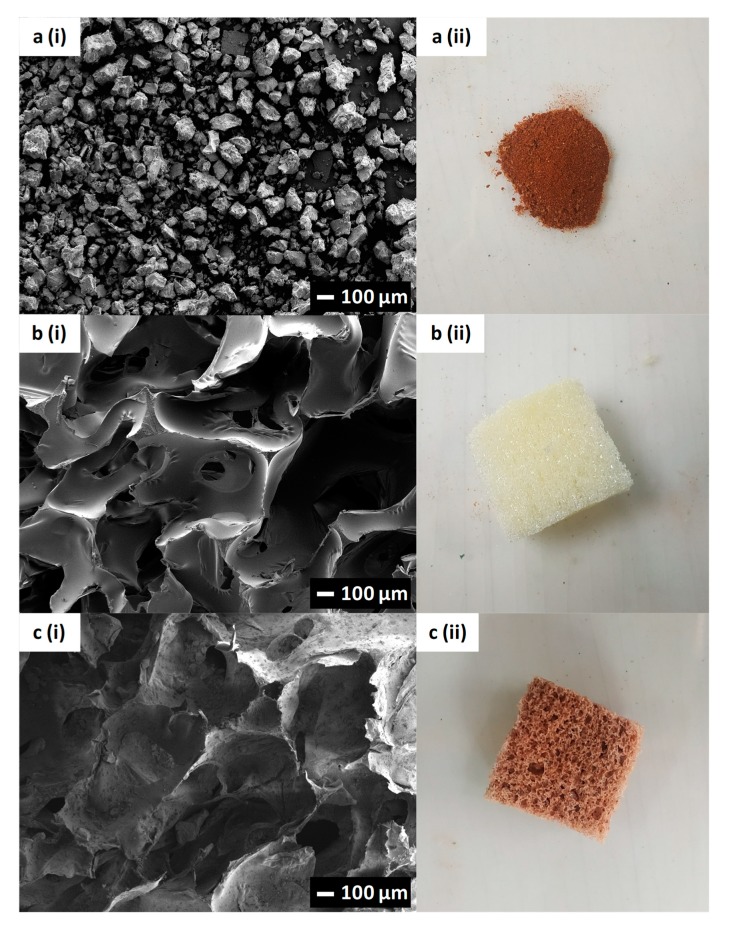
FESEM images and photographs of (**a**) Fe-Si adsorbent; (**b**) PUF; and, (**c**) Fe-Si/PUC.

FESEM-EDX was carried out to study the morphology of Fe-Si, PUF, and Fe-Si/PUC, and to determine the total Fe and Si content in Fe-Si. Figure 3 shows the morphology and photographs of Fe-Si, PUF, and Fe-Si/PUC. The brown Fe-Si (Figure 3(aii)) synthesised was in micro-size with irregular shapes (Figure 3(ai)). Figure 3(bii) and Figure 3(cii) show the photographs of PUF before the impregnation and Fe-Si/PUC, respectively. The PUF was initially pale yellow and changed into brown colour after the impregnation. The FESEM image of PUF (Figure 3(bi)) had a clear surface with large pores. This provides an advantage for the impregnation of Fe-Si onto the surface of the PUF. As shown in the FESEM image of Fe-Si/PUC (Figure 3(ci)), Fe-Si was successfully impregnated onto the PUF surface. Fe-Si can be seen covering the surface of Fe-Si/PUC, as the surface morphology of Fe-Si/PUC is rougher when compared to the PUF. The surface impregnation was successfully carried out by using the proposed method in this paper. Table 1 tabulates the EDX result. The total Fe and Si content of Fe-Si were 14.9% and 26.8%, respectively. The Fe content of the Fe-Si was slightly higher when compared to Fe-Si adsorbent synthesised by Pham and co-workers, 12.3% [11]. It might be caused by calcination of Fe-Si that removed all volatile organic compounds; hence, resulting in a higher Fe content [30].

### 3.4. BET

BET was used to determine the total surface area of Fe-Si. Table 1 presents the total surface area of synthesised Fe-Si. The total surface area of the Fe-Si in the present work was 349 m^2^/g, which is lower as compared to previously reported, 521 m^2^/g [11]. The reduction in total surface area might be contributed by the calcination process, which was not performed by Pham and co-workers. Calcination removed all of the volatile organic group in TEOS, hence reducing the total surface area of Fe-Si [30].

### 3.5. XRD

**Figure 4:**
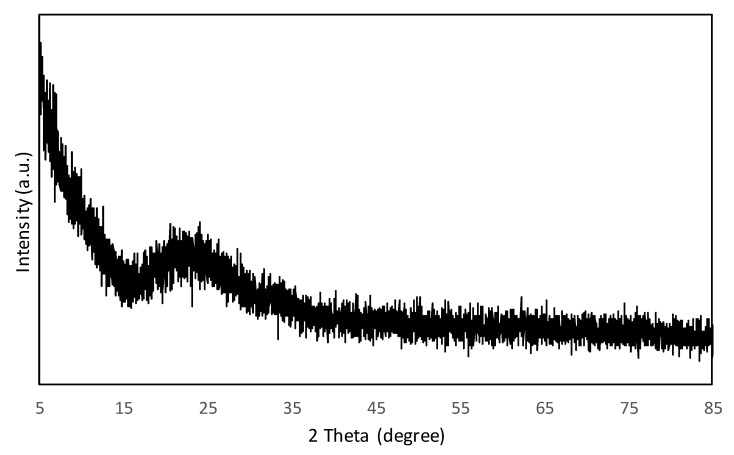
X-ray diffraction (XRD) diffraction pattern of Fe-Si adsorbent.

The XRD diffraction pattern of Fe-Si (Figure 4) shows that the Fe-Si was in an amorphous state, in agreement with previous reports that described the formation of Fe-Si via sol gel method [28,31]. The adsorbent synthesised was composed mainly of Si structure (2θ = 23°). As described in Section 3.3, the EDX results showed that the Si content was almost double the Fe content (Table 1). The peak of Fe crystallites was not observed, probably because of the small amount of Fe in the Fe-Si and inability to be distinguished by XRD.

### 3.6. Water Uptake

Water uptake analysis was carried out to determine the amount of water that can be absorbed by all samples. Table 2 summarises the capability of water uptake and mechanical stability of PUF, PUT, and Fe-Si/PUC. For water uptake, PUT showed a significantly higher amount of water absorbed (566.0%) when compared to PUF (247.0%) and Fe-Si/PUC (340.0%). The treatment of PUF by ethanol increased the capabilities of hydrogen bonding between water and the surface of PUT [32]. Fe-Si/PUC has lower water uptake as compared to PUT because of the Fe-Si impregnation that covered the surface of PUF. Consequently, this reduced the hydrogen binding capabilities and capillary force for water absorption.

### 3.7. Compressive Test Analysis

The mechanical stability of PUF, PUT, and Fe-Si/PUC were tested by using a compressive test. All of the samples were compressed up to 10% of the initial thickness and returned to their original thickness after the compression test. PUT showed the highest compressive stress and modulus (1.90 MPa and 24.43 MPa, respectively), followed by PUF (0.59 MPa and 6.82 MPa, of compressive stress and modulus, respectively) and Fe-Si/PUC (0.23 MPa and 1.08 MPa, of compressive stress and modulus, respectively). Compressive modulus represents the ratio of compressive stress applied as compared to the resulting compression. Samples with high compressive modulus has high flexibility, showing that PUT had the highest flexibility. It was proven that treatment by ethanol improved not only the water absorption, but also the mechanical stability of the foam. However, Fe-Si/PUC had the lowest compressive modulus, hence the lowest flexibility. It can be assumed that the impregnation of Fe-Si onto the surface of the composite caused the Fe-Si/PUC to become more rigid.

### 3.8. TGA/DTG

**Figure 5:**
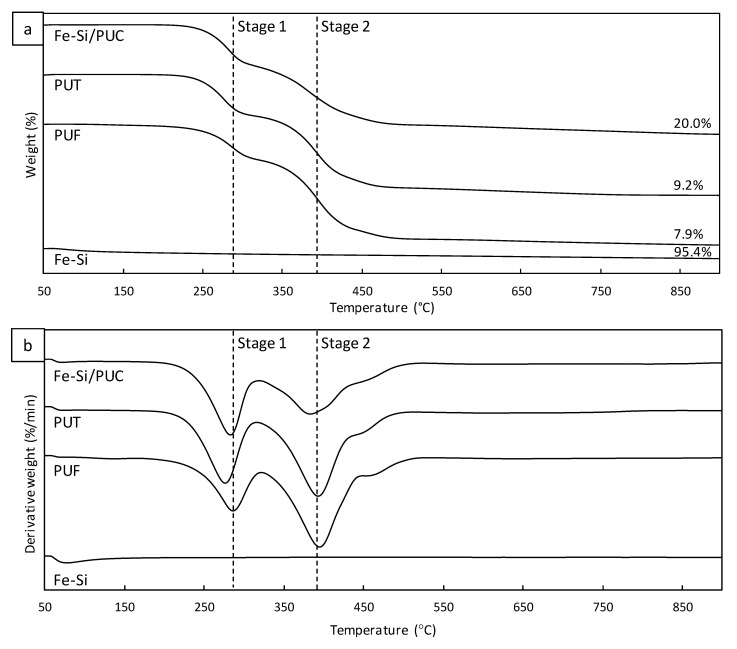
(**a**) Thermogravimetric analysis (TGA) and (**b**) DTG of Fe-Si, PUF, PUT, and Fe-Si/PUC.

TGA/DTG analysis of PUF, PUT, and Fe-Si/PUC showed a similar trend of thermal stability, comprised of two stages of decomposition, as shown in Figure 5a,b. Based on the thermograms, the first stage was observed in the range between 185 °C and 323 °C; attributed to the decomposition of urethane bonds. Fe-Si/PUC, PUT, and PUF experienced 33.8%, 33.4%, and 27.7% of urethane bonds decomposition, respectively. The decomposition of Fe-Si/PUC and PUT were comparable, while PUT had the least. However, the decomposition of urethane bonds in each sample took place at a different temperature range; Fe-SI/PUC from 199 to 321 °C, PUT from 185 to 315 °C, and PUF from 185 to 323 °C. As the PUF went through the highest temperature range of decomposition, it could be assumed that the urethane bonds in PUT and Fe-Si/PUC were more susceptible decomposition. This is also caused by the cleavage of carbonyl double bond during the ethanol treatment. The second stage of decomposition occurred between 320 °C and 534 °C, which could be related to the decomposition of ether group. Fe-Si/PUC, PUT, and PUF experienced 46.7%, 58.1%, and 63.5% of ether group decomposition, respectively. The ether group decomposition temperature range for Fe-Si/PUC, PUT, and PUF were 320 to 517 °C, 315 to 504 °C, and 323 to 534 °C, respectively.

The residue represents the weight of each samples that does not decompose at the temperature 900 °C. Fe-Si/PUC has the highest residue (20.0%), followed by PUT (9.2%) and PUF (7.9%). The 20.0% residue of Fe-Si/PUC was mainly contributed by the amount of Fe-Si immobilised, as Fe-Si is highly thermally stable. It can be observed in the thermogram of Fe-Si, where it remained constant after a decomposition at the range of 60 °C and 370 °C. Approximately 5.0% of decomposition was observed in Fe-Si, which might be contributed by the loss of volatile organic compounds.

### 3.9. Preliminary Study of MB Removal in Aqueous Solution

**Figure 6:**
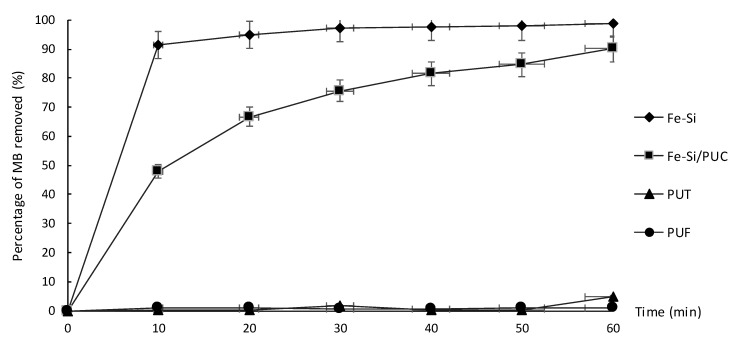
Percentage of methylene blue (MB) removal in aqueous solution (%) against time (min).

Figure 6 shows the result for the removal of MB in aqueous solution. The study of Fe-Si/PUC capability in removing MB at neutral pH aqueous solution was conducted at room temperature and open to the surrounding. Fe-Si/PUC with 0.10 g of Fe-Si was immersed in 100.00 mL of 10 ppm MB. Control experiments were carried out by using PUT, PUF, and Fe-Si as adsorbent in MB solution. The percentage of MB adsorbed by PUF and PUT were 0.8% and 4.9%, respectively. As these values were less than 5%, they could be neglected and assumed to have no significant effect on the MB adsorption by Fe-Si/PUC. Fe-Si adsorbent managed to adsorb 91.3% of MB in the first 10 min., and up to 98.9% at the end of 60 min. This could be related to the total surface area of the Fe-Si adsorbent, as discussed earlier in Section 3.4. The exposed surface area resulted in high MB adsorption over a short period of time. 

**Figure 7:**
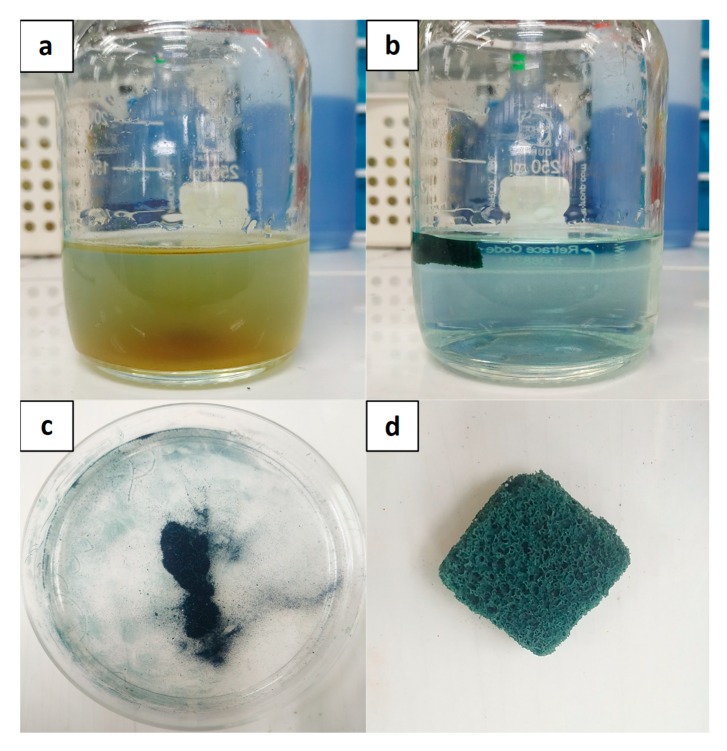
(**a**) Fe-Si and (**b**) Fe-Si/PUC in aqueous MB solution; (**c**) Fe-Si; and, (**d**) Fe-Si/PUC after the recovery process.

On the other hand, Fe-Si/PUC managed to remove MB up to 90.0% at the end of the 60 min. reaction. The embedment of Fe-Si on the surface of PUF had reduced the surface’s active sites of the Fe-Si. However, the performance of Fe-Si/PUC in MB removal was still comparable with powder Fe-Si. Figure 7a,b show the photographs of Fe-Si and Fe-Si/PUC in MB solution, respectively. In Figure 7a, Fe-Si powder dispersed throughout the MB solution and helped in a faster adsorption process. The downside of Fe-Si powder application is some of Fe-Si lost during the recovery process; therefore, Fe-Si/PUC was synthesised by impregnating Fe-Si onto PUF. During the adsorption of MB by using Fe-Si/PUC, there was no Fe-Si powder seen by naked eyes in the reaction solutions, as shown in Figure 7b. Further study was conducted to determine any residual presents and the results are reported in Section 3.11.

The recovery of Fe-Si/PUC was simpler as compared to Fe-Si alone; the whole composite was removed from the reaction system. Figure 7c,d show the Fe-Si and Fe-Si/PUC after the recovery process, respectively. The colour of Fe-Si and Fe-Si/PUC, which were initially brown (Figure 3(aii) and Figure 3(cii), respectively), have turned to blue. The change in colour indicated that MB was adsorbed onto the surface of Fe-Si and Fe-Si/PUC.

### 3.10. Reusability Study of Fe-Si and Fe-Si/PUC

**Figure 8:**
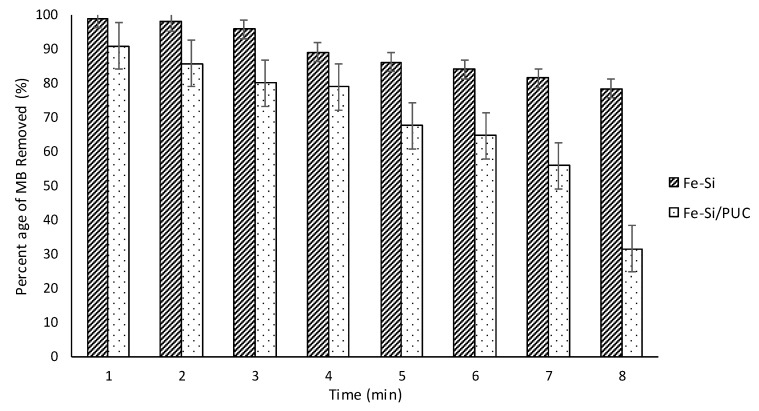
Reusability of Fe-Si and FE-SI/PUC for MB removal in aqueous solution.

Figure 8 shows the reusability study of Fe-Si and Fe-Si/PUC for MB adsorption. It was conducted without desorption to determine the efficiency of the samples in adsorbing MB at a constant concentration repeatedly. It was found that the removal of MB by Fe-Si and Fe-Si/PUC gradually decreased as the number of cycles increased. On the seventh cycle, the performance of Fe-Si in MB removal was considerably high, with the percentage of removal as 81.5%. However, Fe-Si/PUC only removed 55.9%, which is closed to half of the initial MB concentration. The performance of Fe-Si/PUC continued to significantly decrease on the eighth cycle, with only 31.5% of MB being removed, while Fe-Si managed to remove 78.4%. A decrease in available adsorption sites of Fe-Si and Fe-Si/PUC might have caused the gradual decrease in MB removal. Hence, it was concluded that Fe-Si/PUC had lower available active sites for MB adsorption when compared to powder Fe-Si. Based on the reusability study, Fe-Si/PUC could be recycled up to seven times without undergoing desorption process. The recovery process of Fe-Si involved a filtration step and it was found that the amount of Fe-Si recovered decreased as the cycle increased. The stability of Fe-Si/PUC is discussed in detail in Section 3.11.

### 3.11. Quantification of Fe-Si Powder and Fe Residual from Fe-Si/PUC during Reusability Study

TSS and AAS analyses were conducted to determine the concentration of Fe-Si powder and Fe residual from Fe-Si/PUC during the reusability study, respectively. Based on these analyses, the stability of PUF as a support for Fe-Si was evaluated. Table 3 summarises the concentration of Fe-Si powder and Fe residual in MB solution for each adsorption cycle by TSS and AAS, respectively. The analyses were not carried out on MB solution after the eighth cycle, because the composite was no longer capable of removing MB. No trend was observed in the concentration of Fe-Si powder and Fe residual in MB solution throughout the seven cycles. The highest concentration of suspended Fe-Si calculated was 9.10 mg/L (on the third cycle). On the other hand, the highest concentration of Fe residual detected was 0.15 mg/L (on the fifth and sixth cycles). According to the Malaysian Environmental Quality (Industrial Effluent) Regulations 2009, the acceptable conditions for suspended solids and FE in discharged industrial effluent are 50.00 mg/L and 1.00 mg/L, respectively. This composite can be said to be stable, since the highest concentration of suspended Fe-Si and Fe residual that were detected for each cycle were in the accepted range. Thus, this composite itself did not contribute towards secondary pollution.

## 4. Conclusions

This paper proposed a novel yet simple method for Fe-Si adsorbent impregnation onto the surface of PUF to become a composite, namely Fe-Si/PUC. The flexible composite that was produced by this method had high mechanical stability, high capability for water uptake, and high thermal stability. The performance of Fe-Si/PUC in adsorbing MB from aqueous solution was also comparable to the powder Fe-Si adsorbent, with up to 90.0% MB removal in 60 min. The Fe-Si adsorbent recovery was also facilitated, as the PUF support held the adsorbent in place with minimum suspended Fe-Si and Fe residual in the reaction system. This composite could be used up to seven times with 55.9% of MB removal. These properties made Fe-Si/PUC an ideal dye remover for wastewater treatments in the industry. Additionally, Fe-Si/PUC could be considered as an environmentally friendly product that utilised excessive palm oil in Malaysia. The proposed method also offers an exceptional potential in future applications as a catalyst and adsorbent support. Lastly, Fe-Si/PUC possesses a high possibility to be applied in treating other organic pollutants, such as pharmaceutical products.

## Figures and Tables

**Table 1:** Total surface area and total Fe and Si content of Fe-Si.

	BET (m^2^/g)	Fe Content (%)	Si Content (%)
Fe-Si adsorbent	349	14.9	26.8

**Table 2:** Water uptake analysis, compressive stress and modulus of PUF, PUT, and Fe-Si/PUC.

	Water Uptake (%)	Compressive Stress (MPa)	Compressive Modulus (MPa)
PUF	247.0	0.59	6.82
PUT	566.0	1.90	24.43
Fe-Si/PUC	340.0	0.23	1.08

**Table 3:** Concentration of Fe-Si powder and Fe residual in MB solution for each adsorption cycle by TSS and AAS, respectively.

Cycle	1	2	3	4	5	6	7
Fe-Si (mg/L)	3.00	8.80	9.10	1.00	2.10	3.20	1.50
Fe (mg/L)	0.01	0.05	0.08	0.05	0.15	0.15	0.04
